# Mechanical thrombectomy in acute ischemic stroke—experience from 6 years of practice

**DOI:** 10.1007/s00234-014-1353-z

**Published:** 2014-04-01

**Authors:** Åsa Kuntze Söderqvist, Magnus Kaijser, Michael Söderman, Staffan Holmin, Nils Wahlgren, Tommy Andersson

**Affiliations:** 1 Department of Clinical Neuroscience, Karolinska Institutet, Stockholm, Sweden; 2 Department of Neuroradiology, Karolinska University Hospital, 171 76 Stockholm, Sweden; 3 Department of Neurology, Karolinska University Hospital, Stockholm, Sweden

**Keywords:** Mechanical thrombectomy, Ischemic stroke, mRS, NIHSS, Symptomatic hemorrhage

## Abstract

**Introduction:**

We present our results from the first 6 years with mechanical thrombectomy in the treatment of ischemic stroke.

**Methods:**

Every patient treated with mechanical thrombectomy for acute ischemic stroke from September 2005 to December 2011 was consecutively included in this retrospective analysis. Baseline and outcome data were retrieved from computerized records at the hospital. National Institute of Health Stroke Scale (NIHSS) score and the modified Rankin Scale (mRS) score were used as outcome parameters. Favorable outcome was defined as a mRS score of 0–2, corresponding to independence in activities of daily living. We also evaluated revascularization and severe adverse events, with focus on symptomatic intracranial hemorrhage.

**Results:**

Good functional outcome (mRS 0–2) was achieved in 50 % (120/240) of all patients. For patients with no neurological deficit prior to stroke onset (i.e., mRS = 0 before stroke), the proportion with good functional outcome was 54 %. Symptomatic hemorrhages occurred in 4.6 % of the cases (5.7 % in the anterior circulation).

**Conclusion:**

In summary, our results supports that mechanical thrombectomy is a safe and effective method to restore blood flow in selected patients suffering from an acute ischemic stroke.

## Introduction

Recanalization after large vessel occlusion is proven to be an important predictor for favorable outcome in acute ischemic stroke [1, 2]. Intravenous thrombolysis (IVT) with alteplase (recombinant tissue plasminogen activator, rt-PA) has been thoroughly investigated in several randomized controlled studies and is shown to be a reasonably safe and effective treatment [3–8]. However, depending on the time of onset, 5–15 patients have to be treated in order to significantly improve the outcome of one individual [6]. The search for alternative or complementary methods has consequently continued. Mechanical thrombectomy represents such treatment method that has been developed during the last decade [9, 10]. Positive data from mechanical thrombectomy has been published severally in both single and multicenter studies and registries [11–16]. Many of these, however, include a limited number of patients. In this retrospective study, the safety and effectiveness of thrombectomy in the first 6 years of practice in our institution are studied in 240 consecutive patients, representing one of largest cohorts investigated.

## Material and methods

### Clinical setup

Mechanical thrombectomy was initiated at the Karolinska University Hospital in September 2005 until December 2011 in which 240 thrombectomies had been performed. During this 6-year period, endovascular therapy was provided on a 24/7 basis by initially two and eventually four neurointerventionalists at the Department of Neuroradiology, the sole provider of neurointerventional care in the Stockholm area, with a catchment population of more than two million. Every patient treated with mechanical thrombectomy for acute ischemic stroke from September 2005 to December 2011 was consecutively included in this retrospective analysis. Baseline and outcome data were retrieved from computerized patient charts at the hospital, and retrospective image analysis was done through the hospital’s PACS system.

### Patients

Patients were either admitted directly to the Karolinska University Hospital or transferred from one of six referring hospitals within the region. In addition, patients were occasionally sent from more distant hospitals outside the primary catchment area.

All patients arriving from referring hospitals had an unenhanced computed tomography scan (CT) performed before transferring to the Karolinska University Hospital. In the later part of the study, most patients also had a CT angiography (CTA) before transfer. If a large infarction was revealed on the CT scan, with a clear hypodensity exceeding 1/3 of the MCA territory or with extensive brain stem involvement, the patient was not treated with IVT and not transferred to the Karolinska. If the patient was eligible for IVT, the infusion (0.9 mg/kg) was initiated for all patients except for some with basilar artery occlusion, which was in accordance with the stroke protocol at the time. In the early years of the study, i.e., before 2010, the result of the intravenous treatment was awaited, and the patient was re-evaluated after approximately 1 h, then transfer was organized and the patient was sent to the Karolinska. From 2010 and onwards, the patient was instead transferred immediately after initiation of IVT, with ongoing treatment in the ambulance. If a CTA had been performed, an additional prerequisite for transfer was the presence of a large vessel occlusion [principally internal carotid artery (ICA), middle cerebral artery (MCA); segment M1 or M2 or basilar artery (BA)]. Patients that were non-eligible for IVT were transferred if no large infarct was detected and endovascular treatment could be initiated within 8 h after symptom onset. In addition, only patients with a NIHSS score of 8–30 (from 2010 changed to 6–25) without severe comorbidities were considered. Exception was made for a few patients with NIHSS <6 if the symptoms included aphasia. The reason for the upper limit of NIHSS score was that, according to our experience, patients with such high scores also had large manifest infarctions and would gain little from endovascular treatment.

All patients arriving from outside hospitals were transferred directly to the Department of Neuroradiology, thus bypassing the Emergency Room (ER), where representatives for the Departments of Neurology, Anesthesiology, and Neuroradiology met up. A quick neurological examination was performed after which a non-enhanced CT was repeated and complimented with a CT perfusion (CTP) and a CTA. If a CTA had been executed already at the referring hospital, it was repeated only if the patient had dramatically improved clinically.

Patients admitted directly to the Karolinska University Hospital were examined clinically in the ER and brought to the CT scanner with minimal time delay where the same “stroke protocol” was performed (CT, CTA, and CTP). If these patients were eligible for IVT, it was initiated immediately after the scanning.

All patients were subsequently brought directly to the angio suite, some with ongoing infusion of alteplase, where preparation for thrombectomy began. After having read the scans, the decision whether to proceed with thrombectomy or not was made jointly by the stroke neurologist and the neurointerventionist on call. Mechanical thrombectomy was considered for all patients with remaining symptoms, a large vessel occlusion and with no large infarct as interpreted from the CT, CTP, and CTA source images. In order to reduce the risk of reperfusion hematomas, ongoing infusions of alteplase were terminated when a guide catheter had been positioned, and it was likely that the thrombectomy would be technically feasible. The angiography suite preparations were aborted in case of clinical improvement or if the presence of an already large infarct could be determined based on the CT, the CT source images, and the CTP.

The study was approved by the research ethics committee at the Karolinska Institute, and no individual consent was collected before inclusion in the study.

### Thrombectomy technique

The Merci retrievers (Concentric Medical/Stryker Neurovascular, Mountain View, California, USA), including the X5, X6, L5, and L6 devices, as well as the V-series, were used in the beginning of the study period. Since February 2009, different so-called stent retrievers were utilized: Solitaire FR (ev3 Endovascular, Plymouth, MN, USA/Covidien, Irvine, CA, USA), Trevo (Concentric Medical/Stryker Neurovascular), IRIIS/Capture (Mindframe, Irvine, CA, USA/Covidien), and Opticell (MindFrame/Covidien).

For most cases, the standard thrombectomy procedure began with placing the tip of an 80-cm long introducer sheath, mostly an Arrow 8F (Arrow International, PA, USA) in the common carotid artery, just below the bifurcation, in which an 8F Merci balloon guide catheter (Concentric Medical/Stryker Neurovascular) was inserted with its tip positioned in the proximal ICA. In case of a BA occlusion, a 6F Envoy (Cordis Corporation International, Johnson and Johnson Medical NV/SA, Waterloo, Belgium) or a 7F Guider (Boston Scientific, Natick, Massachusetts, USA/Stryker Neurovascular) was most of the time used as guide catheter.

After confirming the presence of the thromboembolus on an angiogram, a microcatheter was advanced through the thrombus with the aid of a microguidewire. After release of the stent retriever, centered at the obstructive blood clot, it was left in place for approximately 5 min (3–10) before the actual thrombectomy was performed. The balloon was inflated with simultaneous aspiration in the guide catheter to create flow reversal in the ICA. For early cases in the series, where the Merci device was used, the device was positioned distal to the thromboembolus.

For patients with so-called tandem occlusions in the anterior circulation, i.e., a proximal carotid severe stenosis or occlusion due to acute dissection or atherosclerosis, in addition to the MCA occlusion, the procedure was modified to include angioplasty or carotid stenting as well as to sometimes contain the use of an intermediate catheter, mostly the so-called “distal access catheter” (DAC; Concentric Medical/Stryker Neurovascular).

For a few patients, the underlying cause for the large vessel occlusion was an intracranial stenosis. Also, for these patients, the standard procedure was altered to include angioplasty and sometimes stenting. Placement of a stent, extra- or intracranially, was, however, avoided when possible due to the need for anti-aggregation, which in the context of a potential acute ischemic infarct may impose a risk for a severe reperfusion hematoma. If a stent was inserted, the patient received half the recommended bolus dose (0.125 mg/kg body weight) of abciximab (Reo-Pro; Eli Lilly, Sweden), but no infusion, followed by acetylsalicylic acid (Trombyl; Pfizer AB, Sollentuna, Sweden; bolus of 300 mg and then 75 mg daily for a minimum of 6 months) and clopidogrel (Plavix; Bristol-Myers Squibb AB, Bromma, Sweden; bolus of 300 mg and then 75 mg daily for a minimum of 3 months) after approximately 24 h when a routine follow-up CT scan did not reveal a significant hematoma.

As a supplement to the thrombectomy, for treating small distal emboli, alteplase was sometimes infused intra-arterially together with careful microcatheter and microguidewire manipulations. The dose of alteplase was maximized to 10 mg in case the patient had received IVT, whereas up to 20 mg could potentially be administered if no IVT had been given. Other adjunctive therapies included 2–5 mg of intra-arterial nicardipine (Cardene; PDL BioPharma Inc., Fremont, CA, USA) during the procedure if there was a tendency to focal vasospasm, and in the beginning of the series, acetylsalicylic acid (bolus of 300 mg and then 75 mg daily) and low molecular weight heparin (dalteparin sodium, Fragmin; Pfizer AB, 2,500 IU twice daily) were used after the procedure in non-stented patients to avoid re-occlusion. For patients later in the series (from 2010), usually, no anti-aggregation or low molecular weight heparin was administered post-procedure since re-occlusion in the absence of a stent was found not to be a significant problem.

### Baseline and outcome data

Baseline and outcome data were retrieved from computerized records at the hospital. National Institute of Health Stroke Scale (NIHSS) score [17] and the modified Rankin Scale (mRS) score [18] were used as outcome parameters. A stroke neurologist assessed the NIHSS score before and after each intervention. In case NIHSS score pre- or post-treatment was not specifically noted in the records, but the criteria was described in the text, we assessed the score based on the text. In four cases of anterior circulation stroke, it was not possible to access enough information from the charts to assess a pre-treatment score, and in 13 patients, it was impossible to establish a post-treatment score. Information on the mRS score was for the majority (59 %) of patients obtained at follow-up 3 months after the procedure. For a minority, the scoring was postponed until 6 months post-treatment and for 9 % of the cases, it was delayed even more until maximum of 12 months after the thrombectomy procedure. The mRS scoring was done by an independent stroke neurologist or by a stroke research nurse certified for such evaluations, who also, in a few cases with missing mRS score estimated the value from the description in the charts. Favorable outcome was defined as a mRS score of 0–2, corresponding to independence in activities of daily living.

All the angiographies performed at the time of thrombectomy were re-evaluated by two of the authors (ÅKS and TA) to obtain definitive information on pre- and post-procedure mTICI score (thrombolysis in cerebral infarction) [19, 20]. The mTICI score was defined as follows: 0 = no perfusion; 1 = penetration, but no distal branch filling; 2a = perfusion with incomplete (<50 %) distal branch filling; 2b = perfusion with incomplete (≥50 %) distal branch filling; and 3 = full perfusion with filling of all distal branches. A post-procedure mTICI score of ≥2b was considered to represent a successful revascularization. One of the authors (ÅKS) also re-examined all pre- and post-procedure unenhanced CT scans to obtain ASPECTS scores (Alberta Stroke Program Early CT Score) [21] and to determine the presence of any post-interventional hemorrhage. Hemorrhagic transformation was classified into hemorrhagic infarction (HI) type 1 and 2 and parenchymal hematoma (PH) type 1, type 2, and remote parenchymal hematoma (PHr-2) in accordance with the ECASS definitions [22]. We defined symptomatic intracranial hemorrhage according to the SITS-MOST definition: a PH-2 (blood clot exceeding 30 % of the infarcted volume with significant space occupying effect) or subarachnoid hemorrhage on the post-treatment imaging scan leading to a decline in NIHSS of ≥4 points or causing death (mRS 6) within 36 h [23].

For the statistical analysis, we used SAS statistical software (V.9.1; SAS Institute Inc.). Ninety-five percent confidence intervals (95 % CI) were calculated assuming a binomial distribution. For testing independence between outcome and exposure variables, we used Pearson’s exact chi-squared tests. Observations with missing values were excluded. A *p* value <0.05 was considered statistically significant.

Since a delayed assessment of mRS score may lead to an overestimation of patients with good outcome, we assessed the potential impact of the delayed mRS assessments by doing sensitivity analyses where an extra point was added to the mRS score for all patients who were assessed after more than 6 months.

Test for trends were done by analyzing categories as continuous variables in logistic regression using the PROC LOGISTIC statement in SAS™.

## Results

### Baseline data

Baseline data about the patients are presented in Table 1. During the study period, 240 patients were selected for thrombectomy, and of these, 192 had an anterior circulation occlusion. A more detailed presentation on the results on patients with stroke in the posterior circulation has been presented elsewhere [24], and we therefore focus on the anterior circulation. Fifteen of the patients in the study were previously included in the multicenter study by Davalos et al. [25], all of them had anterior circulation strokes.

**Table 1:** Patient characteristics by thrombus location and pre-intervention IVT

		Anterior and posterior circulation		Anterior circulation					
				All		IV tPA		No IV tPA	
		nr	%	nr	%	nr	%	nr	%
All		240	100.0	192	100.0	89	100.0	103	100.0
Sex	Male	138	57.5	104	54.2	50	56.2	54	52.4
	Female	102	42.5	88	45.8	39	43.8	49	47.6
Age	16–49	38	15.8	29	15.1	15	16.9	14	13.6
	50–64	70	29.2	56	29.2	22	24.7	34	33.0
	65–79	110	45.8	89	46.4	46	51.7	43	41.7
	80–xx	22	9.2	18	9.4	6	6.7	12	11.7
mRS baseline	0	202	84.2	161	83.9	79	88.8	82	79.6
	1	26	10.8	21	10.9	7	7.9	14	13.6
	2	9	3.8	7	3.6	3	3.4	4	3.9
	3	2	0.8	2	1.0	0	0.0	2	1.9
	4	1	0.4	1	0.5	0	0.0	1	1.0
Time to Karolinska	Within 3 h	95	39.6	80	41.7	38	42.7	42	40.8
	3–4.5 h	45	18.8	34	17.7	27	30.3	7	6.8
	More than 4.5 h	33	13.8	25	13.0	14	15.7	11	10.7
	In hospital	32	13.3	26	13.5	4	4.5	22	21.4
	Wake up	28	11.7	22	11.5	4	4.5	18	17.5
	Missing	7	2.9	5	2.6	2	2.2	3	2.9
iv rtPa		96	40.0	89	46.4	89	100.0	0	0.0
NIHSS pre-thrombectomy (median = 16)	0–5			6	3.1	1	1.1	5	4.9
	6–11			39	20.3	17	19.1	22	21.4
	12–19			91	47.4	48	53.9	43	41.7
	20–35			52	27.1	22	24.7	30	29.1
	Missing			4	2.1	1	1.1	3	2.9
pre-ASPECT	0–5			29	15.1	18	20.2	11	10.7
	6–7			57	29.7	29	32.6	28	27.2
	8–9			72	37.5	35	39.3	37	35.9
	10			32	16.7	7	7.9	25	24.3
	Missing			2	1.0	0	0.0	2	1.9
Time to groin puncture	Within 3 h	27	11.3	23	12.0	9	10.1	14	13.6
	3–4.5 h	59	24.6	50	26.0	25	28.1	25	24.3
	More than 4.5 h	87	36.3	66	34.4	45	50.6	21	20.4
	In hospital	32	13.3	26	13.5	4	4.5	22	21.4
	Wake up	28	11.7	22	11.5	4	4.5	18	17.5
	Missing	7	2.9	5	2.6	2	2.2	3	2.9
mTICI pre-thrombectomy	0	219	91.3	180	93.8	83	93.3	97	94.2
	1	10	4.2	6	3.1	4	4.5	2	1.9
	2a	6	2.5	3	1.6	0	0.0	3	2.9
	2b	2	0.8	1	0.5	1	1.1	0	0.0
	Missing	3	1.3	2	1.0	1	1.1	1	1.0

There were 138 (57.5 %) men and 102 (42.5 %) women; 45 % of the patients were under the age of 65, 46 % between 65 and 79, and 9 % were ≥80 years old. The median NIHSS score was 16 (16 for the anterior circulation and 11 for the posterior circulation). Ninety-six patients (40 %) were treated with IVT before the endovascular intervention. Baseline mRS, i.e., the functional status of the patients before the present stroke, was one or more in 18 % of the cases. In two patients, the procedure failed, as it was impossible to enter the intracranial circulation; in one case due to severe tortuosity of the carotids and in the other due to an ICA dissection, both reported as having a missing mTICI score. In one patient, it was impossible to report ASPECT before the intervention because no CT was performed as the patient was transported straight from the operating room to the angiography table because of distal MCA (M2) thromboembolus after MCA bifurcation aneurysm surgery. Two hundred nineteen (91 %) of the occlusions were graded as mTICI 0 before the intervention, i.e., a complete occlusion of that vessel. There was no significant difference in baseline characteristics between patients treated and not treated with IVT.

### Radiological and clinical outcome

The radiological and clinical results of the thrombectomies are presented in Table 2. Seventy-two percent of the patients had their final angiography runs graded as mTICI ≥ 2b (successful recanalization) and 7 % as mTICI 0 (no recanalization).

**Table 2:** Number and proportion of post-thrombectomy recanalization (mTICI score), NIHSS score, NIHSS change, and modified Rankin Scale score (mRS) by thrombus location and pre-intervention IVT

		Anterior and posterior circulation		Anterior circulation					
				All		IV tPA		No IV tPA	
		nr	%	nr	%	nr	%	nr	%
All		240	100.0	192	100.0	89	100.0	103	100.0
mTICI post-thrombectomy	0	15	6.3	13	6.8	6	6.7	7	6.8
	1	9	3.8	6	3.1	2	2.2	4	3.9
	2a	42	17.5	33	17.2	17	19.1	16	15.5
	2b	88	36.7	67	34.9	30	33.7	37	35.9
	3	84	35.0	72	37.5	33	37.1	39	37.9
	Missing	2	0.8	1	0.5	1	1.1	0	0.0
NIHSS post-thrombectomy (median = 8)	0–5			81	42.2	33	37.1	48	46.6
	6–11			35	18.2	17	19.1	18	17.5
	12–19			42	21.9	23	25.8	19	18.4
	20–35			21	10.9	10	11.2	11	10.7
	Missing			13	6.8	6	6.7	7	6.8
NIHSS change (median = −6)	≥12			46	24.0	20	22.5	26	25.2
	−11 to −4			75	39.1	32	36.0	43	41.7
	−3 to +3			41	21.4	22	24.7	19	18.4
	> +4			16	8.3	9	10.1	7	6.8
	Missing			14	7.3	6	6.7	8	7.8
mRS at 3 months	0	16	6.7	12	6.3	5	5.6	7	6.8
	1	49	20.4	43	22.4	20	22.5	23	22.3
	2	55	22.9	40	20.8	17	19.1	23	22.3
	3	48	20.0	40	20.8	21	23.6	19	18.4
	4	27	11.3	25	13.0	12	13.5	13	12.6
	5	8	3.3	4	2.1	2	2.2	2	1.9
	6	35	14.6	26	13.5	12	13.5	14	13.6
	Missing	2	0.8	2	1.0	0	0.0	2	1.9

During the study period, NIHSS was not generally assessed for patients with posterior circulation strokes. We therefore present data on NIHSS for anterior circulation strokes only. The NIHSS score improved by 4 or more points in 63 % of the patients during the primary care, with NIHSS pre- or post-intervention missing in 7.3 % of the cases.

Good functional outcome (mRS 0–2) was achieved in 50 % (120/240) of all patients. The figure for the subgroup of patients with an anterior circulation stroke was almost identical (49 %, 95/192). Finally, 47 % (42/89) of patients that had received IVT before the endovascular procedure had a good functional outcome compared to 51 %(53/103) of those that did not receive IVT. For patients with no neurological deficit prior to stroke onset (i.e., mRS = 0 before stroke), the proportion with good functional outcome was 54 %.

### Serious adverse events

*Device-related problems* were accounted in six patients, possible to solve in five of those cases, but in one patient, the pusher wire broke, the device was left in place, and a carotid stent had to be inserted to hold the wire in place and avoid migration (Table 3). It did not cause any clinical symptoms, but added an extra risk for the patient due to the need of anti-aggregation treatment.

**Table 3:** Adverse events by thrombus location and pre-intervention IVT

		Anterior and posterior circulation		Anterior circulation					
				All		IV tPA		No IV tPA	
		nr	%	nr	%	nr	%	nr	%
All		240	100.0	192	100.0	89	100.0	103	100.0
Complications	Device problems	6	2.5	5	2.6	3	3.4	2	1.9
	Dissection	7	2.9	6	3.1	4	4.5	2	1.9
	Perforation	8	3.3	8	4.2	2	2.2	6	5.8
	Air emboli	1	0.4	1	0.5	0	0.0	1	1.0
Hemorrhage	HI1	14	5.8	13	6.8	6	6.7	7	6.8
	HI2	11	4.6	11	5.7	7	7.9	4	3.9
	PH1	11	4.6	11	5.7	7	7.9	4	3.9
	PH2	12	5.0	12	6.3	7	7.9	5	4.9
	PHr1	0	0.0	0	0.0	0	0.0	0	0.0
	PHr2	1	0.4	1	0.5	1	1.1	0	0.0
	SAH/intraventr.	24	10.0	23	12.0	13	14.6	10	9.7
Vasospasm		19	7.9	15	7.8	9	10.1	6	5.8
Symptomatic hemorrhage		11	4.6	11	5.7	7	7.9	4	3.9

*Procedure-related complications* were encountered in 16 patients. Dissections were caused in seven of those, six of which did not lead to any clinical deterioration. In one case, there was a suspicion of excessive force during the thrombectomy causing damage to the lenticostriate arteries, leading to a mortal intracerebral hemorrhage. In eight patients, a vessel perforation occurred during the procedure, one of them located in the common iliac artery. Of the remaining seven intracranial vessel perforations, two caused mortal hemorrhages. Two other cases were treated by parent vessel occlusion with coils, not causing any increase in NIHSS score. The remaining three intracranial perforations did not need any further endovascular treatment nor did they cause any clinical deterioration. In one patient, an air emboli was seen on the angiogram, but it did not cause any clinical symptoms. In total, out of 16 procedure-related complications, only three cases resulted in clinical deterioration or death.

Small hemorrhages, not causing any mass effect, were commonly seen, but such small bleeds never resulted in a detectable clinical deterioration. Eight patients (4.2 %), all of them having the obstruction in the anterior circulation, suffered from a symptomatic intracerebral hemorrhage according to the SITS-MOST definition [8]. These hemorrhages were located in the infarcted territory (so-called “reperfusion hematomas”), most of which occurred a few hours after the procedure. In addition, three hemorrhages were caused during the procedure as described above, resulting in a total rate of symptomatic intracranial hemorrhage of 5.7 % in the anterior circulation (4.6 % in total). For patients treated with IVT prior to thrombectomy, there was more than twofold increase in risk for symptomatic hemorrhage, but this increase in risk was not statistically significant (OR 2.1, 95 % CI 0.60–7.47). In this study, no symptomatic hemorrhage was seen in patients ≥80 years old, and there were no significant associations between the incidence of symptomatic hematomas and sex, age, baseline mRS, pre-interventional NIHSS and ASPECT scores, or time between disease onset and treatment.

### Difference between thrombectomy devices

We analyzed mTICI score post-thrombectomy, mRS at follow-up, and serious adverse events by different endovascular recanalization methods (Table 4). Non-stent retrievers alone were used in 47 patients. Most of them were variants of the Merci retriever, but in a few cases, the Amplatz snare was used. In 151 patients, stent retrievers were used, and in 15 cases, we used a combination of these techniques. In 16 cases, no thrombectomy device was used, but instead, intra-arterial thrombolysis was performed alone or in combination with mechanical manipulation with microcatheter and guidewire and/or aspiration. In five patients, no endovascular treatment was performed at all since it was not possible to reach the intracranial circulation (due to severe tortuosity or dissection) or not possible to pass the thrombus with the microcatheter. In one patient, the thrombus had dissolved by the time of the first contrast injection during angiography.

**Table 4:** Outcome (mTICI post-thrombectomy and mRS) and selected adverse events by endovascular recanalization method

		Total		Non-stent retriever^a^		Stent retrievers^b^		Combination^c^		IAT mm^d^		No intervention^e^	
		nr	%	nr	%	nr	%	nr	%	nr	%	nr	%
All		237	100.0	47	100.0	151	100.0	15	100.0	16	100.0	6	100.0
mTICI post-thrombectomy	0	15	6.3	1	2.1	3	2.0	1	6.7	5	31.3	5	83.3
	1	9	3.8	3	6.4	5	3.3	0	0.0	1	6.3	0	0.0
	2a	41	17.3	13	27.7	23	15.2	3	20.0	2	12.5	0	0.0
	2b	88	37.1	19	40.4	54	35.8	7	46.7	7	43.8	1	16.7
	3	84	35.4	12	25.5	67	44.4	4	26.7	1	6.3	0	0.0
mRS at 3 months	0	16	6.8	1	2.1	11	7.3	2	13.3	2	12.5	0	0.0
	1	49	20.7	6	12.8	38	25.2	2	13.3	2	12.5	1	16.7
	2	54	22.8	12	25.5	34	22.5	1	6.7	5	31.3	2	33.3
	3	48	20.3	14	29.8	25	16.6	3	20.0	4	25.0	2	33.3
	4	27	11.4	2	4.3	18	11.9	4	26.7	2	12.5	1	16.7
	5	8	3.4	1	2.1	5	3.3	1	6.7	0	0.0	1	16.7
	6	35	14.8	11	23.4	20	13.2	2	13.3	1	6.3	1	16.7
Complications	Device problems	6	2.5	0	0.0	6	4.0	0	0.0	0	0.0	0	0.0
	Dissection	6	2.5	2	4.3	4	2.6	0	0.0	0	0.0	0	0.0
	Perforation	8	3.4	2	4.3	5	3.3	1	6.7	0	0.0	0	0.0
Vasospasm		19	8.0	1	2.1	17	11.3	1	6.7	0	0.0	0	0.0
Symptomatic hemorrhage		11	4.6	4	8.5	7	4.6	0	0.0	0	0.0	0	0.0

^a^Non-stent retrievers: Merci (48), Amplatz (1). The figures do not add up because of more than one retriever being used in the same patient

^b^Stent retrievers: Solitaire (107), Trevo (23), IRIIS (20), Opticell (13), and Capture (9)

^c^Combination of stent and non-stent retrievers

^d^IAT alone or in combination with mechanical manipulation with microcatheter and guidewire and/or aspiration

^e^No intervention performed. In five cases, it was not possible to reach the intracranial circulation or pass the thrombus, and in one case, the thrombus had dissolved

**Fig 1:**
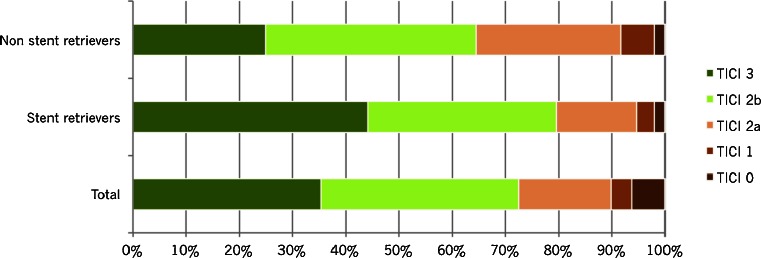
mTICI score post-thrombectomy, total (237 patients), stent retrievers (151 patients), and non-stent retrievers (mainly Merci) (47 patients)

**Fig 2:**
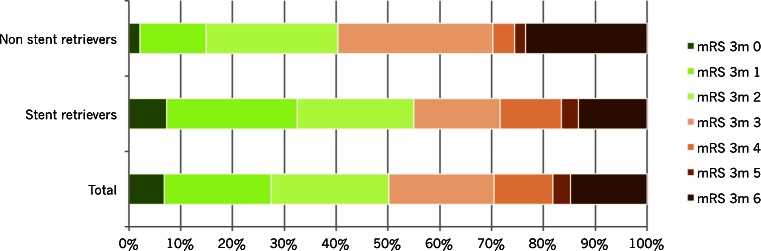
mRS scale score 3 months post-thrombectomy, total (237 patients), stent retrievers (151 patients), and non-stent retrievers (mainly Merci) (47 patients)

**Fig 3:**
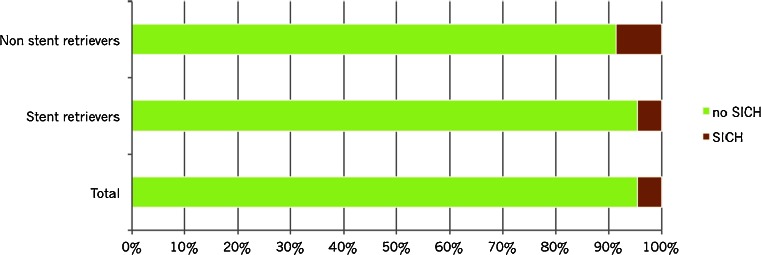
Symptomatic intracranial hemorrhage (SICH) post-thrombectomy, total (237 patients), stent retrievers (151 patients), and non-stent retrievers (mainly Merci) (47 patients)

The proportion of patients with high mTICI scores post-thrombectomy and low mRS scores at follow-up was higher with stent retrievers than with non-stent retrievers (Figs. 1 and 2), and also, the incidence of symptomatic hemorrhage (Fig. 3) and dissections was lower when stent retrievers were used, but the differences between stent, non-stent, and the combination of both were not statistically significant. The incidence of device problems and vasospasm was higher with the stent retrievers. There were no statistically significant differences in outcome or serious adverse events when extra- or intracranial stenting was performed.

## Discussion

In this study, we found that when performing mechanical thrombectomy for ischemic stroke in the anterior circulation, neither prior treatment with intravenous thrombolysis nor high-attained age was significantly associated with risk for symptomatic intracranial hemorrhage. The proportion of all treated patients with favorable outcome after thrombectomy was 50 %, and 54 % when restricting the analysis to patients with no neurological deficits prior to stroke onset.

We found that by treating acute occlusions of the large cerebral vessels with mechanical thrombectomy, we could restore flow in 72 % of the patients (mTICI ≥2b), and 50 % of all patients had gained independence (mRS 0–2) at follow-up. In cases where we used only stent retrievers, mTICI ≥2b was established in 80 % compared to 65 % when non-stent retrievers were used (no statistical difference). In a recent retrospective multicenter study of patients treated with the Solitaire FR device [25], mTICI ≥2b was established in 85 %. Even so, there was no difference in the rate of independent patients (54 vs 55 %) at follow-up between that and the present study. In the SWIFT trial [26], Solitaire was compared with the Merci Retrieval System. A TIMI score [27] of 2 or 3 was regarded as a successful recanalization achieved in 61 % of the Solitaire FR-treated patients, but only in 24 % of the patients treated with Merci. In spite of this relatively low percentage of revascularization success, also for the Solitaire FR, 58 % of the patients treated with that device reached a mRS score of 0–2 at 3 months follow-up. In a third recent study comparing the TREVO device with Merci [11], the results were slightly different as compared to SWIFT. In this TREVO 2-study, recanalization defined as mTICI ≥2b was seen in 86 % of the stent retriever treated patients, but only 40 % of them reached independence (mRS 0–2) after 90 days. A higher independence rate (55 %) was seen in the TREVO EU study [28] with the same device although with a slightly lower recanalization rate (78.3 %). Importantly, this study included almost half of the patients within 3 h (onset to groin puncture) while the corresponding delay in the first TREVO 2 study was 4.7 h. In our study, several different devices were used (Solitaire, TREVO, Opticell, IRIIS, and Merci), more or less from the starting point of their European approval.

Symptomatic hemorrhages occurred in 4.6 % of the cases (5.7 % in the anterior circulation). This proportion is higher than the 1.8% SICH reported for patients in the SITS-MOST registry [23]. The patients in our study, however, had a median NIHSS score of 16 compared to 12 in the SITS-MOST database, and 36.3% of patients were treated more than 4.5 h after onset of symptoms compared to less than 1 % in SITS-MOST [23, 29]. Since it has been shown that both pre-treatment NIHSS score and time to treatment are important risk factors for SICH [30], it is likely that the high proportion of SICH in our study at least in part is due to more severely ill patients. None of the symptomatic hemorrhages occurred in the oldest patient group (>80 years). Patients receiving intravenous therapy before the mechanical thrombectomy procedure did not have a significantly higher rate of symptomatic hemorrhages compared to patients that did not receive IVT. In consequence, mechanical thrombectomy seems safe to perform even in old patients as well as in those receiving intravenous fibrinolytic therapy.

Strengths of the present single-center study include study size and the consistency by which the acute stroke patients were selected and treated. Other strengths were the possibility to have access to updated medical records of all patients, and that all consecutive patients were included in the study. Even though protocol changes in the patient flow were made during the study period in order to decrease time delay and treat patients faster, the principles of patient selection and basic treatment techniques remained intact.

There are three limitations in the study. Firstly, since the study was not based on patients selected according to predefined criteria, but on all patients referred to the department, the study is hampered by missing data. For example, for some patients, there are missing data on post-interventional NIHSS score (7 %). To address this problem, we treated all patients with missing information on post-interventional NIHSS score as non-successful thrombectomies. This approach reduces the risk of over-interpreting the benefits of the procedure. For missing information on mRS score after 3 months, we used the information gathered not after 3 months but after six or more months instead. This may increase the proportion of patients with favorable outcome. Reassuringly though, adding an extra point to the mRS score of all the patients with mRS assessed after more than 6 months only marginally altered the results. The second limitation is that this study emanates from a single center, limiting the possibility to generalize the results. Thirdly, the observational design of the study is subject to all limitations inherent with this design.

In summary, our results supports that mechanical thrombectomy is a safe and effective method to restore blood flow in selected patients suffering from an acute ischemic stroke. Further prospective, multicenter studies comparing mechanical thrombectomy to IVT that may confirm our results are ongoing.
